# A Question of Criminal Intent

**Published:** 1911-09

**Authors:** 


### A Question of Criminal Intent

At the present time there are four physicians of this city under criminal charges before the City court of Buffalo as a result of the activities of the Erie County Medical Society. The charges against these practitioners are definite and evidence, carefully prepared, is being presented against one of them to prove that he is guilty of the crime of attempting abortion.

The physicians under arrest are Monroe Manges, Martin Willoughby, Arthur Collins and Frederick Vogt. Dr. Manges is being examined in the city court and is being defended by an able attorney who is using every technicality in the intricacies of the law to free his client of blame. The complainant in all the cases is a woman detective employed by the Erie County Medical Society to gather evidence against suspects and from the testimony thus far presented her plan of procedure has been carried out and apparently she has secured sufficient evidence on which the police acted without hesitation. The woman detective was assisted by a male companion.

There is a co-defendant of Manges, a woman who kept a rooming house on Franklin street where the detective took an apartment and arranged for her aborting. Dr. Manges, it is testified, came to the house in response to a call from the woman who kept the house—he had a patient in the house at the time—in the morning and without seeing the detective stated that he would return in the afternoon and make an examination. He did call later with instruments and he was paid $40 according to previous agreement, $15 of which was given to the rooming housekeeper. He then prepared for the "examination" of the patient, and was about to begin the examination when detectives came from an adjoining room and arrested him. A physician was called in for the purpose of examining the instruments displayed and his evidence will be introduced to show that they were not wholly those used for making an examination. The cases of Drs. Collins, Vogt and Willoughby have been presented, the prisoners arraigned and admitted to bail. Their examinations will be held later when it is determined regarding the points raised by Manges' attorney who contends that because the detective admittedly was not pregnant it were impossible for the physician to be guilty of the crime of attempted abortion; that one might as well be charged with "killing a corpse." To this the district attorney answers that a pickpocket could not be considered guiltless of attempt to pick a pocket because the pocket happened to be empty. These be tangled questions of law trimmed of technicality with which the courts alone have to deal; yet they are interesting and call for definite decision. In the present case they will be unquestionably cleared up before the ending of the case for it is the expressed intention of Manges' attorney to carry his case to the Court of Appeals if necessary. And it is quite likely the other cases will be permitted to rest until it is decided whether an abortion may be attempted in the absence of a pregnancy. The law considers intent in criminal cases and it may be that these Buffalo cases will be the means of establishing a precedent in abortion trials. Certainly the matter should be cleared up with a finality that would make unnecessary the juggling which accompanies the trial of so important a phase of criminal practice.

Heretofore the difficulty in obtaining convictions in such cases has been the lack of corroborative evidence on the part of the woman who has been aborted either through her disappearance or her disinclination to face a jury and tell the story of her moral delinquency. With a clear interpretation of the intent in such cases the hucksters of the profession will find it difficult to peddle their wares.

The Erie County Medical society is following the trail blazed by the New York County society apparently fearlessly and let us hope, successfully. There be none of vengeance in these cases. The prosecution is merely complaining and presenting evidence to sustain its contention that the men under arrest agreed to perform an operation forbidden by the law of this state; and broadly considered, the law holds that a person agreeing and consenting to commit a felony is guilty of the commission of a crime even though the specific act be not committed. There is no sentimental phase of this question at all viewed through legal eyes or professional eyes. If the men under arrest are guilty they should be punished. If the law says they are innocent they should be reinstated to their professional standing. At the same time it is the duty of the prosecution to determine the question of intent in these cases and so have an end to legal quibbling and Scotch verdicts. N. W. W.

